# Deep-learning-based endoscopic single-shot fringe projection profilometry

**DOI:** 10.1117/1.JBO.30.8.086003

**Published:** 2025-08-19

**Authors:** Ruizhi Zuo, Shuwen Wei, Yaning Wang, Ruichen Huang, Wayne Wonseok Rodgers, Jinglun Yu, Michael H. Hsieh, Axel Krieger, Jin U. Kang

**Affiliations:** a Johns Hopkins University, Department of Electrical and Computer Engineering, Baltimore, Maryland, United States; b Johns Hopkins University, Department of Mechanical Engineering, Baltimore, Maryland, United States; c Children’s National Hospital, Division of Urology, Washington, DC, United States; d Johns Hopkins University, School of Medicine, Baltimore, Maryland, United States

**Keywords:** 3D optical imaging, fringe projection profilometry, endoscope, deep learning, surgery guidance

## Abstract

**Significance:**

Conventional fringe projection profilometry (FPP) requires multiple image acquisitions and therefore long acquisition times that make it slow for high-speed dynamic measurements. We propose and demonstrate a deep-learning-based single-shot FPP system utilizing a single endoscope for surgical guidance.

**Aim:**

We aim to achieve real-time depth map generation of target tissues with high accuracy for robotic surgical guidance.

**Approach:**

We proposed an endoscopic single-shot FPP system based on a deep learning network to generate real-time accurate tissue depth maps for surgical guidance. The system utilizes a dual-channel endoscope, where one channel projects fringe patterns from a projector and the other channel collects images using a camera. In addition, we developed a data synthesis method to generate a large number of diverse training datasets. The network consists of MaskNet, which segments the tissue from the background, and DepthNet, which predicts the depth map of the image. The results from both networks are combined to generate the final depth map.

**Results:**

We tested our algorithm using fringe patterns with different frequencies and found that the optimal frequency for single-shot FPP in our setup is 20 Hz. The algorithm has been tested on both synthetic and experimental data, achieving a maximum depth prediction error of ∼2  mm and a processing time of about 12.75 ms per frame.

**Conclusion:**

A deep-learning-based single-shot FPP endoscopic system was shown to be highly effective in real-time depth map generation with millimeter-scale error. Implementing such a system has the potential to improve the reliability of image-guided robotic surgery.

## 1 Introduction

Fringe projection profilometry (FPP) is a camera-based three-dimensional (3D) optical imaging technique that uses structured light illumination to generate a dense and accurate depth map and 3D point cloud. Because of its precision, we have been developing FPP as a machine vision system for robotic surgery.^1^^–^^4^ In this context, the surgical robot utilizes point cloud data to plan, control, and adjust its task trajectory, enabling autonomous surgery.^1^^,^^2^ When combined as a laparoscopic imaging system, FPP can be used to guide minimally invasive surgery, which is increasingly preferred in modern clinical practice for their ability to minimize surgical trauma and expedite recovery.^3^^,^^4^ Conventional FPP techniques use a projector to project a set of sinusoidal fringe patterns with multiple frequencies and phase shifts on the target, where the surface depth information is naturally encoded into the images captured by a camera at a different view from the projector. After retrieving the phase of the captured images using a phase unwrapping algorithm, a depth map can be determined, and the corresponding point cloud can be reconstructed.^5^ However, the need to capture multiple images in FPP results in slow measurement speeds, at around 7 frames per second (FPS),^3^ and is prone to motion distortion in living tissue.^6^ Moreover, the required synchronization of the camera and projector adds to the system’s complexity.

To accelerate the speed and therefore enable dynamic measurements based on FPP, integrating a single-shot endoscopic fringe image and a deep learning scheme for accurate depth reconstruction is proposed and demonstrated in this work. In prior work, U-Net^7^ has been demonstrated as an end-to-end network to obtain a depth map utilizing a single-shot fringe-pattern grayscale image^8^ or a red-green-blue (RGB)^9^ image as input. Relatedly, Wang et al.^10^ have used a generative adversarial network (GAN) architecture, i.e., pix2pix, as this can also be considered to be an image translation problem. A transformer-based model has also been reported to be an end-to-end depth prediction method.^11^ Conversely, another approach is to combine deep learning with the conventional FPP algorithm. Yang et al.^12^ and Yu et al.^13^ have used deep learning to extract the phase information from an input fringe pattern image. A constraint-based phase unwrapping network has also been proposed for FPP,^14^ from which the depth is then calculated. Nguyen and Wang^9^ have developed networks using single-shot images to generate multiple images with different frequencies and phases and then reconstructed the depth map with a conventional workflow. However, though most of these methods have been evaluated on synthetic data^15^ to showcase the concept, the real clinical data are more challenging because the image backgrounds are noisier and more intricate. Furthermore, these approaches have mainly concentrated on the depth reconstruction algorithm, with a limited focus on hardware considerations. Optical designs, such as those employing separate endoscopes for projection and imaging,^16^ are complex and heavy, which reduce the system’s usability in clinical settings. In addition, most fringe-based techniques use sinusoidal pattern projections. The sinusoidal pattern provides finer phase information than the binary patterns,^17^^–^^19^ thus a more accurate depth reconstruction. However, a well-designed sinusoidal pattern requires a complex optical setup, such as fiber-optic interference^20^^,^^21^ or a single, expensive mask.^22^ In addition, the precise detection of the phase information of the pattern depends heavily on the dynamic range of the camera and the associated optical system.

In this work, an endoscopic single-shot FPP system with a modified two-path neural network depth reconstruction algorithm was developed and demonstrated. First, we designed a dual-channel endoscope optical setup to image the intestinal phantom samples. The system’s compact design improves its deployment efficiency and integrability with a robotics surgical system. Second, we developed a depth reconstruction neural network having two paths: one for segmenting the sample from the background (MaskNet) and the other for predicting the depth map (DepthNet). The results from both paths were combined to produce the final depth reconstruction result. The algorithm was evaluated on both the synthetic dataset and intestinal phantom samples. Third, the method was tested using both sinusoidal and binary patterns, and the results indicated that binary patterns yield better reconstruction outputs. This conclusion suggested that a single-shot FPP system can be achieved using binary patterns generated from a low-cost mask with high accuracy.

## 2 Method

### 2.1 Overview

**Fig. 1:**
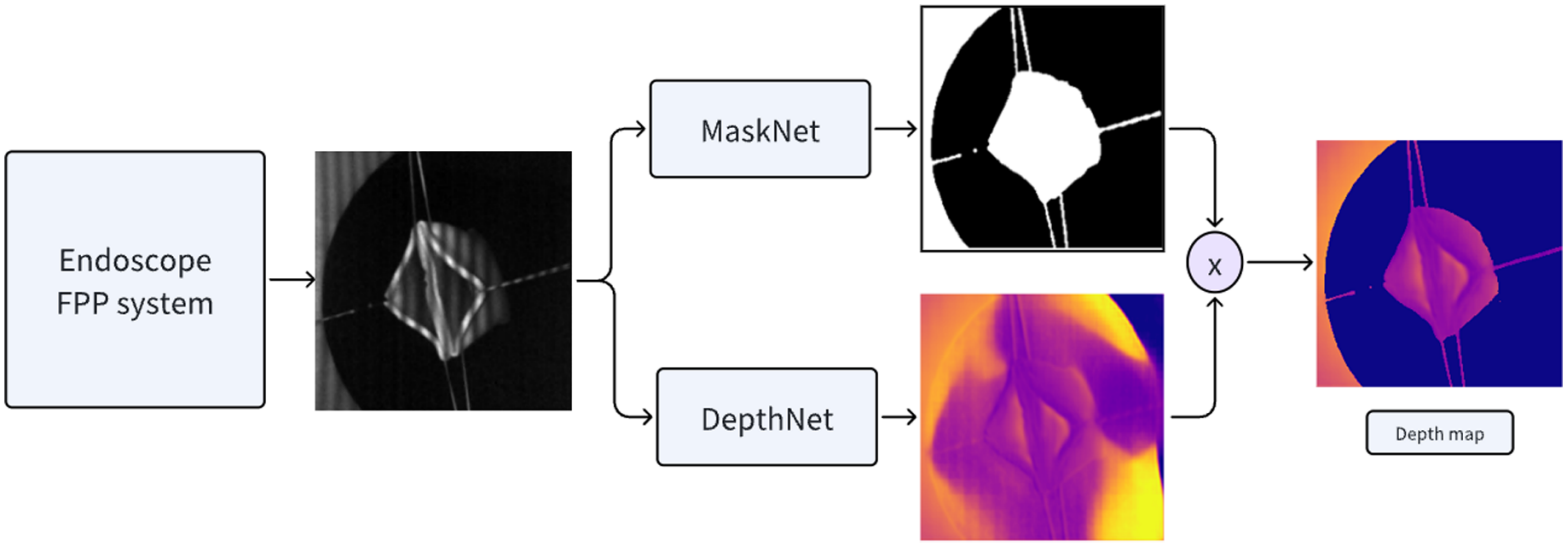
Workflow of the deep-learning-based endoscopic FPP system: the pattern-distorted image acquired from the endoscopic FPP system is input into both MaskNet and DepthNet. The segmentation output from MaskNet and the depth prediction from DepthNet are combined via pixel-wise multiplication to produce the final depth map.

Figure 1 illustrates the workflow of the proposed method. First, we developed a single-shot endoscope FPP system to capture pattern-projected images of two cut ends of the intestine during the creation of an intestinal anastomosis. To assess and optimize network performance, we also implemented a data synthesis program to generate additional training datasets. The captured images were fed into two networks: MaskNet segmented the sample from the background, and DepthNet predicted the sample’s depth map. The results from both networks were then combined via pixel-wise multiplication to generate the final depth map. In addition, corresponding point clouds were created for surgical guidance.

### 2.2 Endoscope FPP System

**Fig. 2:**
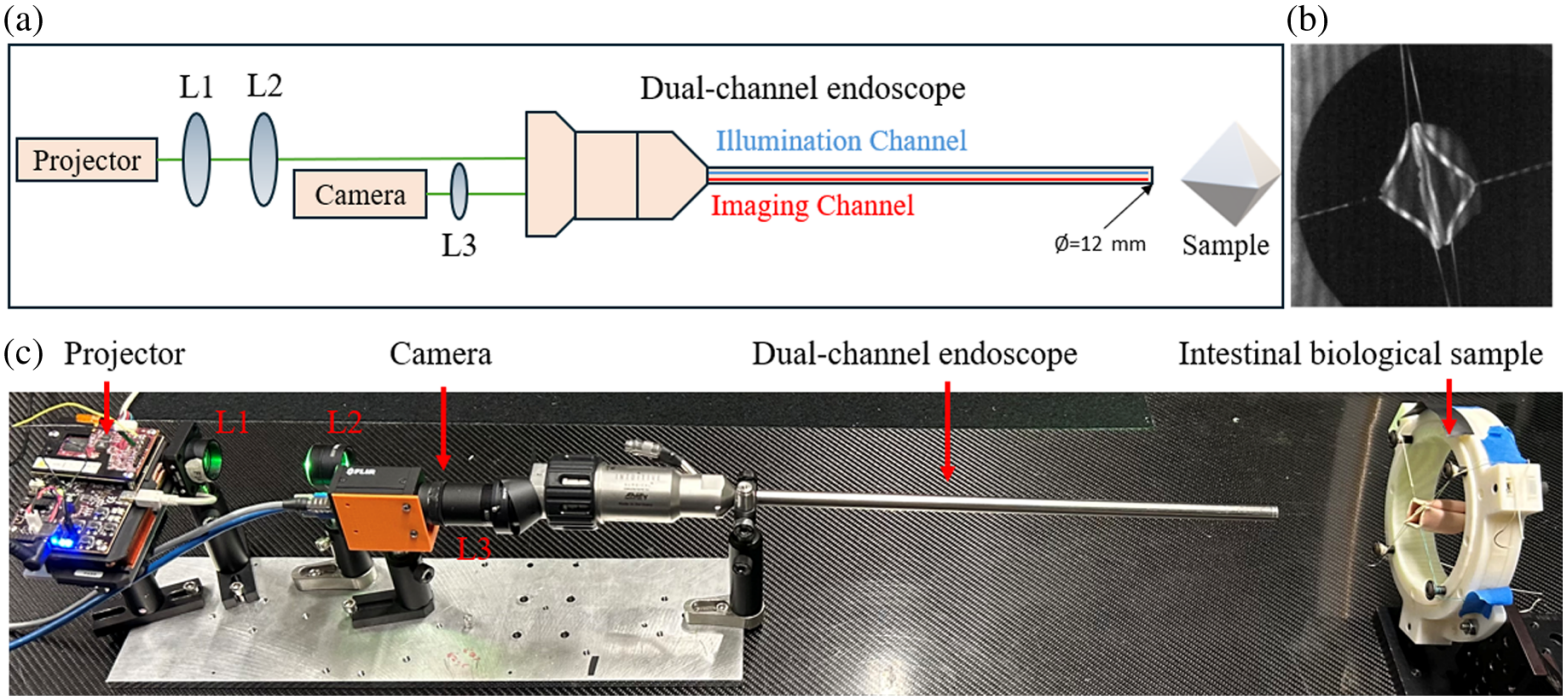
(a) Schematic representation of the endoscope FPP system optical setup. L1 to L3, lenses. (b) Grayscale image acquired from the camera. (c) Photograph of the optical system setup.

The endoscope FPP system is illustrated in Fig. 2(a). The fringe pattern was generated from a projector (DLP3010EVM-LC, Texas Instruments). The projector has an output resolution of 1280×720  pixels, which enables a clear phase gradient in the projected patterns. We selected the green channel with high intensity to enhance contrast in surgical scenarios, where the scene is often dominated by red tones. The light from the projector was collimated by two lenses (L1: AC254-030-A-ML f=30  mm, L2: AC254-075-A-ML f=75  mm, Thorlabs) and was guided by the illumination channel of the dual-channel endoscope (311464-05, Intuitive). The distorted patterns on sample surfaces were imaged through the imaging channel of the dual-channel endoscope and focused onto a camera (GS3-U3-51S5M-C FLIR) by an imaging lens (L3: LA1608, f=75  mm, Thorlabs) as shown in Fig. 2(b). The camera and projector were synchronized using the programmable interface and a custom-designed circuit to ensure that image acquisitions are accurately aligned with the projected patterns. We chose an intestinal phantom tissue as a sample. The sample was pulled into a diamond shape using strings, with the four corners selected as landmarks to guide the surgical robotic arm during intestine anastomosis procedures.^16^ Figure 2(c) shows a photograph of the optical setup.

### 2.3 Data Synthesis Program

In the single-shot setup, several parameters need to be optimized. The most important parameter is the frequency of the fringe pattern. Lower frequencies provide more accurate phase measurements but are less sensitive to sample details, whereas higher frequencies are more sensitive to sample details but less accurate for phase measurement in the sample. Thus, finding an optimal frequency for the single-shot FPP system is essential. In addition, the difference in reconstruction accuracy between sinusoidal and binary patterns is also investigated. Due to the long acquisition time of the FPP system and the limited surgical data set, we had a limited amount of training data. To increase the training data size and diversity, we developed a data synthesis program based on Blender, an open-source 3D creation software, to efficiently generate the training data with different projection parameters for the model.

**Fig. 3:**
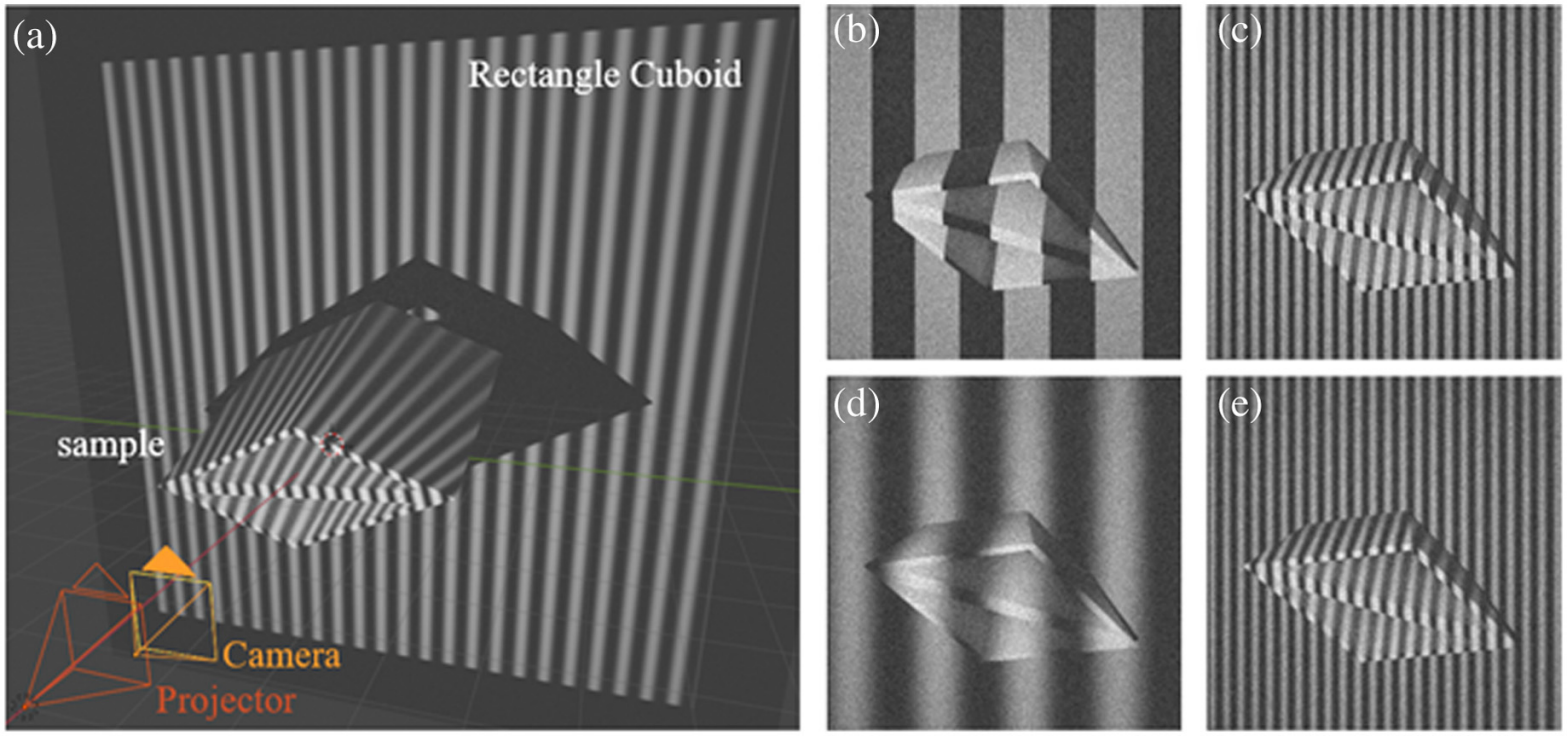
(a) Schematic of the data synthesis program. The projector and camera are placed in front of the diamond-shaped sample. The sample has a random position and rotation during data acquisition. A rectangular cuboid is placed behind the sample to avoid the background being infinite. We show some examples generated from the synthesis program with binary pattern frequencies of (b) 4 Hz and (c) 24 Hz, and with sinusoidal pattern frequencies of rows (d) 4 Hz and (e) 24 Hz.

Figure 3(a) shows the simulation setup we built in Blender. We created a diamond-shaped sample object to simulate intestinal samples as observed in Fig. 2(b). The projector was positioned in front of the sample to project the different sinusoidal or binary patterns with various frequencies. The distances between the projector, camera, and sample tissue were configured to replicate the real setup. The camera was used to capture the image of the sample with random positions, orientations, and distortions. Because Blender did not provide ray-tracing-based optical modeling or a dedicated lens module, the camera lens was manually set to be 30 mm in Blender to ensure that the magnification closely matches that of the physical system. The output image resolution was also configured to match that of the actual endoscopic system. However, as a simulation environment, Blender did not account for physical optical aberrations or sensor noise. To better mimic real-world imaging conditions, Poisson noise was added to the generated images during post-processing. The sample, with an original side length of 4 cm, underwent random translations (−1 to 1 cm), rotations (−15  deg to +15  deg), and scaling factors (0.85 to 1.15) along all three axes. A large rectangular cuboid was placed behind the sample to avoid the background being infinite. The software had built-in functionality to generate the depth map for every image.

For each sample at a certain fixed translation, rotation, and scaling, we collected 22 grayscale images as a group, with frequencies ranging from 4 to 24 Hz with a 2-Hz increment, for both binary and sinusoidal patterns. To model the noise during image acquisition, we added noise based on the photon shot noise characteristics inherent to optical imaging systems. Specifically, we used the Poisson distribution to simulate the shot noise stochastic nature of photon arrival at the sensor. The noise for each pixel was not assigned a fixed value; instead, it is calculated individually based on the pixel intensity, ensuring that regions with lower intensity are affected by higher noise levels. This approach allowed us to realistically replicate the spatially varying noise observed in practice, thereby validating the feasibility and robustness of our method under representative imaging conditions. The mean peak signal-to-noise (PSNR) between the noisy images and the original images was 27.56. Figure 3 shows sample image data for binary patterns of the frequency of (b) 4 Hz and (c) 24 Hz and for sinusoidal patterns of (d) 4 Hz and (e) 24 Hz.

### 2.4 Data Preparation

The network was first trained on the synthetic dataset using both sinusoidal and binary patterns at different frequencies to obtain the optimized pattern parameters. For each pattern, we generated 1200 pairs of pattern-projected images and corresponding depth maps with a size of 256×256. We split the data into a training set of 800, a validation set of 200, and a testing set of 200. The ground truth for MaskNet is easily generated because the background is located much deeper than the sample.

After determining the optimal pattern, we used it for collecting the real dataset and trained the network. We collected 307 groups of images by manually adjusting the sample’s position and orientation. We used 250 for training, 27 for validation, and 30 for testing. The original size of the image was 1024×1024. Initially, we employed a Gaussian filter with a window size 11×11 and a standard deviation of 2 to denoise the image.

Each group included 18 images: 16 images ($I_{1}∼I_{16}$) with 4 frequencies (1, 4, 16, 64 Hz) and 4 phase shifts ($0,\frac{\pi }{2},\;\pi ,\frac{3\pi }{2}$) that were used to generate the depth map (D) from the conventional FPP algorithm. In addition, the remaining two images in sinusoidal ($I_{S}$) and binary ($I_{B}$) were utilized for training. The depth map (D) was computed by

$$z=\frac{c_{0}+c_{1}\phi +(c_{2}+c_{3}\phi )u+(c_{4}+c_{5}\phi )v}{d_{0}+d_{1}\phi +(d_{2}+d_{3}\phi )u+(d_{4}+d_{5}\phi )v},$$ (1)

where z is the depth of the sample, $c_{0∼5}$ and $d_{0∼5}$ are the system calibration parameters,^23^ (u,v) are pixel index, and ϕ is unwrapped phase calculated from the image set ($I_{1}∼I_{16}$).^16^ For MaskNet, the binary ground truth (M) was generated by calculating the pixel variance of the images $I_{1}∼I_{16}$ using the formula below,

$$M_{i}=\{\begin{array}{cc} 0, & \mathrm{Var}(I_{1i},I_{2i},\ldots I_{16i})<\gamma ,\\ 1, & \mathrm{Var}(I_{1i},I_{2i},\ldots I_{16i})\geq \gamma , \end{array}$$ (2)

in which i represents the pixel index in the image and γ denotes the variance threshold value. The background is typically located farther from the sample, resulting in lower reflectivity compared to the biological tissue sample. This makes pixel intensity in the background less sensitive to the projection pattern phase, leading to lower intensity variance. γ is set to 30 in our case, but it can vary depending on the optical setup. Next, morphological opening and closing operations were applied to remove small noise and fill small holes in the mask image to generate the final mask. All images used for training $\left(I_{S},I_{B},D,M\right)$ were downsampled to 256×256 to ensure consistency with the synthetic data and to accelerate training.

### 2.5 Network Architecture

The proposed architecture consisted of two networks: MaskNet and DepthNet. MaskNet segmented the sample from the background, and DepthNet predicted the depth map. The outputs of both networks were multiplied to produce the final depth map of the sample.

**Fig. 4:**
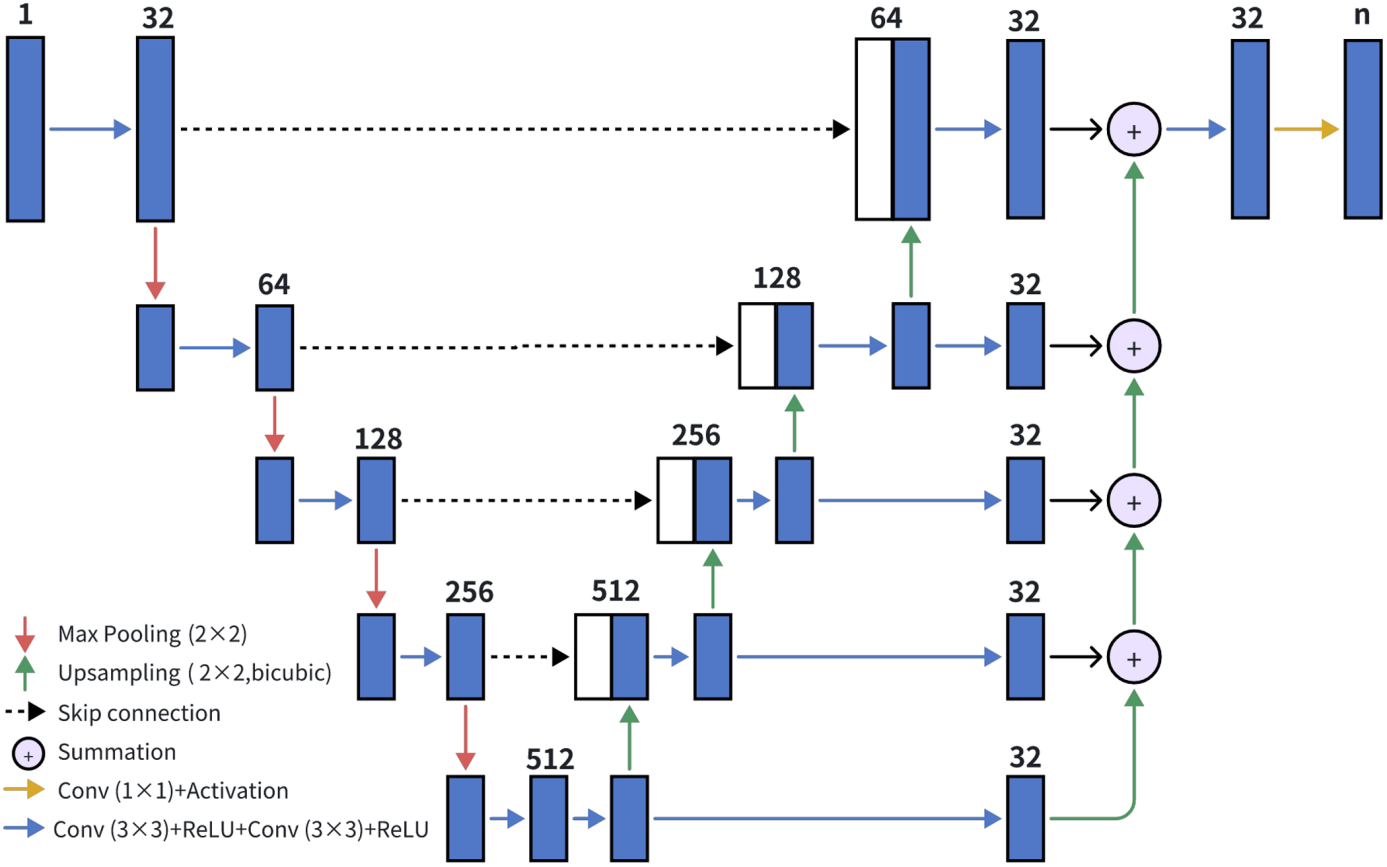
Network architecture of the MaskNet and DepthNet. The network is a modified U-Net with multilevel information. The MaskNet used softmax as an activation function with n=2, and the DepthNet used sigmoid as an activation function with n=1.

Both MaskNet and DepthNet were based on a modified five-level U-Net, as shown in Fig. 4, which leverages the advantage of multiresolution analysis. The blue arrows represent convolutional layers with a 3×3 window size, followed by a rectified linear unit (ReLU) activation function. The red arrows represent max pooling operations, with a fixed window size. Zero-padding is applied to all convolution layers to prevent image cropping. The green arrows indicate 2×2 bicubic upsampling operations, whereas the black dotted arrows represent skip connections from the encoder to the decoder. Unlike the conventional U-Net architecture, our multilevel network extracts information directly from the different levels of the decoder and sums them to generate the final output. It has been demonstrated that the multilevel information in U-Net can accelerate network convergence and improve output accuracy.^24^ The yellow arrows represent convolutions with a 1×1 window size, followed by an activation function with n-channel output. For MaskNet, we applied the softmax function as the activation, with a 2-channel output. For DepthNet, the sigmoid function was used as the activation to limit the output range, with a single output layer as depth prediction.

### 2.6 Loss Function

MaskNet and DepthNet were trained separately with different loss functions. The trainable parameters of the utilized networks were optimized based on our depth reconstruction loss function $L_{d}$ and mask segmentation loss function $L_{m}$.

#### 2.6.1 Depth reconstruction loss

The output of DepthNet ranges from 0 to 1 due to the sigmoid activation. To align this output with physical distance, we applied a linear function. For instance, in our endoscope setup, the distance between the endoscope and the sample ranges from 70 to 140 mm. If we define the output of DepthNet as d∈(0,1), the final depth prediction ($\tilde{D}$) is given by the equation $\tilde{D}(d)=f(d)=150\times d+30$ in millimeters ensuring the prediction covers the real-world distance. To ensure the network focuses on sample prediction, we applied a mask to the depth map during loss calculation, preventing background depth predictions from influencing the training process.

We used the depth reconstruction loss to calculate the difference between the predicted depth values ($\tilde{D}$) and the ground truth (D) with the assistance of a mask (M), which included $L_{1}$ loss and structured similarity index measure (SSIM)^25^ loss as follows:

$$L_{d}(\overset{˜}{D},D)=\alpha \cdot {\left\|\overset{˜}{D}⊗M-D⊗M\right\|}_{1}+(1-\alpha )\cdot \frac{1-\mathrm{SSIM}(\overset{˜}{D}⊗M,D⊗M)}{2},$$ (3)

in which α=0.85 is the weighting parameter and ⊗ denotes the pixel-wise multiplication. The first term in $L_{1}$ loss was used to minimize the overall error, and the second term was used to calculate the SSIM loss between the prediction and ground truth to optimize the sample structure.

#### 2.6.2 Segmentation loss

We used Dice loss^26^ as the segmentation loss function due to its effectiveness in handling the class imbalance between background and sample regions in the images. We define the prediction result of MaskNet as $\tilde{M}$, and the segmentation loss is noted as follows:

$$L_{m}(\tilde{M},M)=1-\mathrm{DSC}(M,\tilde{M})=1-\frac{1}{C}\sum_{j}^{C}\frac{\sum_{i}^{N}2M_{ij}{\tilde{M}}_{ij}+\beta }{\sum_{i}^{N}M_{ij}+\sum_{i}^{N}{\tilde{M}}_{ij}+\beta },$$ (4)

in which DSC denotes the Dice score, and C is the total number of classes of the image, j is the class category index, i denotes the pixel index, and N is the total number of pixels. In our case, we had one class of background and one class of sample, and we set C=2. It is common to add a β factor to both the numerator and denominator for numerical stability. We set β=0.001 in the network training.

### 2.7 Training Details

The proposed method was implemented using the PyTorch^27^ framework. The network was trained for 150 epochs with a multistep decay learning rate schedule. The initial learning rate was set to 0.0001, with decay milestones at epochs 20 and 60, each applying a decay factor of 0.2. Training was conducted using the Adam^28^ optimizer on an NVIDIA RTX A2000 GPU with 12 GB VRAM.

## 3 Results

To demonstrate the performance of the proposed method, we evaluated it using both synthetic data and experimental data collected from the endoscope FPP system. For the quantitative analysis, we used three metrics introduced by Eigen et al.,^29^ which have been widely employed for the performance evaluation of depth estimation: Mean absolute error (MAE), absolute relative error (Abs Rel), and accuracy. They are defined as follows:

$$\mathrm{MAE}=\frac{1}{\left|V\right|}\underset{i\in V}{\sum }|Y_{i}-{\overset{˜}{Y}}_{i}|,$$ (5)

$$\mathrm{Abs}\;\mathrm{Rel}=\frac{1}{\left|V\right|}\underset{i\in V}{\sum }\frac{\left|Y_{i}-{\overset{˜}{Y}}_{i}\right|}{\overset{˜}{Y_{i}}},$$ (6)

$$\text{Accurancy}=\text{percentage of}\;Y_{i}\;\mathrm{s.t.}\;\max(\frac{Y_{i}}{{\overset{˜}{Y}}_{i}},\frac{{\overset{˜}{Y}}_{i}}{Y_{i}})<\delta ,$$ (7)

in which Y=D⊗M and $\overset{˜}{Y}=\overset{˜}{D}⊗\overset{˜}{M}$ denote the final depth map from the ground truth and the network prediction, respectively. i is the index of the pixels. $V=\{i\in \{0,1,\ldots ,N-1\}|M_{i}=1\}$ represents the set of valid pixels, defined as the pixels belonging to the sample. |V| denotes the number of elements in V. Background pixels are excluded to avoid skewing the model’s assessment, as a high proportion of background pixels could negatively impact the evaluation. δ is the thresholding value set for accuracy.

We evaluated the MaskNet using the Dice score and mean intersection over union (mIoU). The IoU is defined by dividing the number of overlapping pixels (intersection) by the total number of pixels that belong to either the predicted ($\tilde{M}$) or ground truth (M) regions for that class (union). The formula is written as

$$\mathrm{IoU}=\frac{M\cap \overset{˜}{M}}{M\cup \overset{˜}{M}}.$$ (8)

The IoUs of all 2 classes (background and tissue in our case) were averaged to compute the mIoU.

### 3.1 Validation Using Synthetic Data

**Fig. 5:**
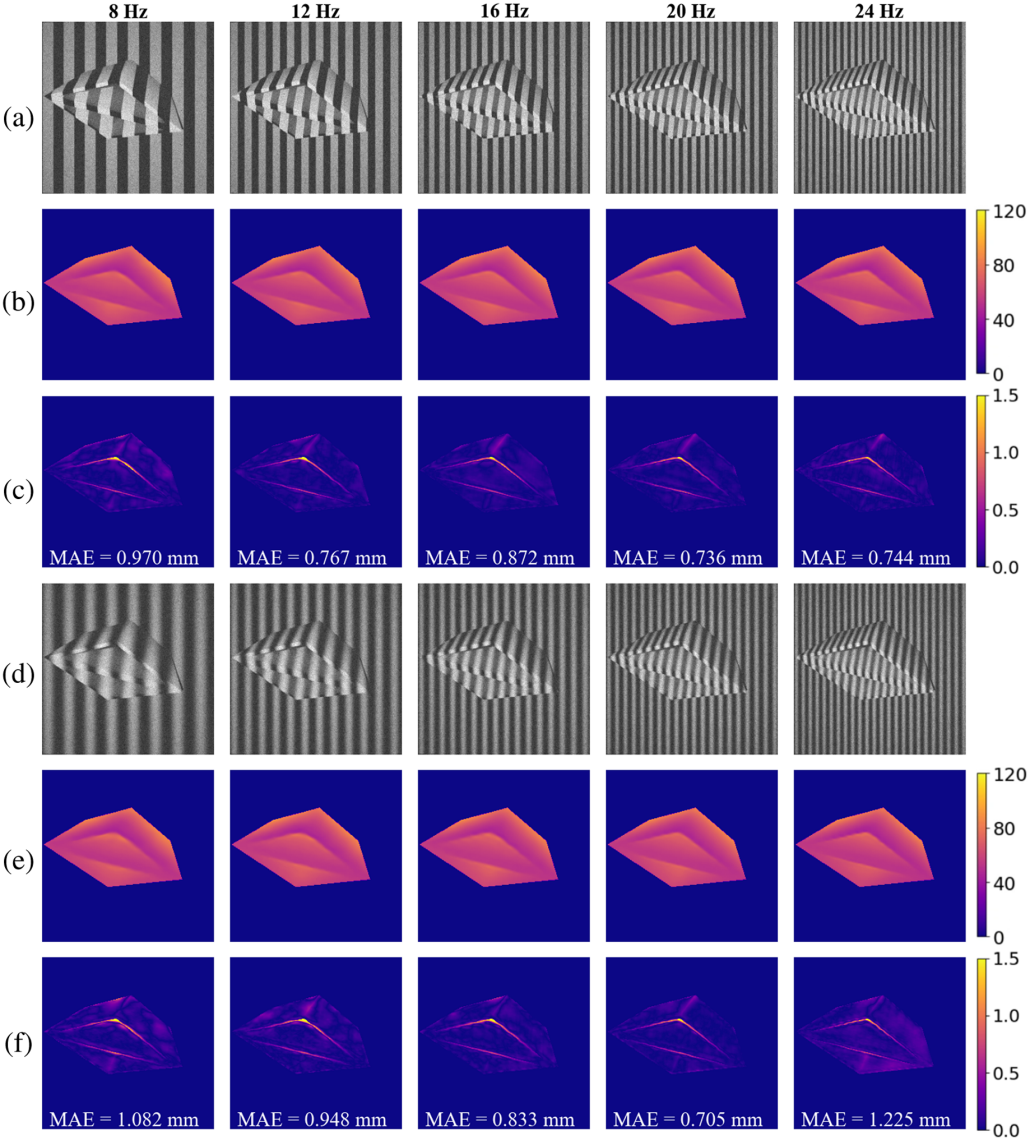
Depth reconstruction result and error map of synthetic data with pattern frequencies of 8, 12, 16, 20, and 24 Hz. Rows (a) and (d) are the original images with binary patterns and sinusoidal patterns, respectively. Rows (b) and (e) are the corresponding depth reconstruction results, and rows (c) and (f) are the corresponding absolute error maps, respectively (depth unit: millimeter).

The synthetic dataset with two patterns and multiple frequencies was used for training, and a selection of depth reconstruction results is presented. We chose 8, 12, 16, 20, and 24 Hz as examples, which are shown in Fig. 5. In this figure, the original images with binary patterns, the corresponding depth reconstruction results, and the absolute error maps are shown in rows (a), (b), and (c), respectively. The original images with sinusoidal patterns, the corresponding depth reconstruction results, and the absolute error maps are shown in rows (d), (e), and (f), respectively. The MAE is displayed on each error map to quantitatively indicate the error level. Note that the image with 20 Hz shows the least error and produces better results on smooth surfaces in both patterns. The error primarily occurs at the sharp edges of the sample but does not affect our system’s ability to detect the sample’s corners.

**Fig. 6:**
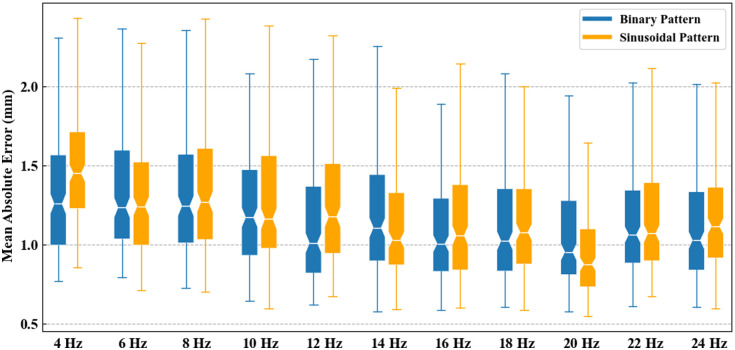
MAE of depth reconstruction from the synthetic dataset with (blue) binary pattern and (yellow) sinusoidal patterns.

We used MAE as a metric to quantitatively analyze the relationship between depth reconstruction and frequency, as shown in Fig. 6. For both binary and sinusoidal patterns, the best result shows a similar trend, with the optimal result occurring at 20 Hz. This can be explained by the fact that images with lower pattern frequencies cannot accurately detect shape details due to the low phase gradient. Conversely, higher frequencies often result in aliasing problems because of the finite sampling rate of the camera, leading to ambiguities in surface and depth reconstruction. Thus, 20 Hz appears to be a compromise between low and high frequency quantification effects.

A detailed quantitative evaluation of the synthetic dataset, using both depth prediction and segmentation metrics, is presented in Table 1. For images with binary patterns, the optimal values for each metric range from 16 to 24 Hz, but the differences are minimal. Moreover, the best results for sinusoidal patterns consistently occur at 20 Hz. Therefore, we conclude that for our setup, 20 Hz should be considered the optimal frequency for single fringe patterns to generate the depth map.

**Table 1:** Quantitative evaluations on the synthetic dataset with different patterns and frequencies. Bold values represent the best performance in each metric.

Patterns		Depth metrics				Segmentation metrics	
Type	Freq (Hz)	MAE ↓	Abs Rel ↓	δ=1.1 ↑	$\delta =1.1^{2}$ ↑	DSC ↑	mIoU ↑
Binary pattern	4	1.35	0.0227	0.9677	0.9922	0.9944	0.9740
	6	1.39	0.0231	0.9636	0.9907	0.9954	0.9781
	8	1.37	0.0231	0.9687	0.9922	0.9976	0.9885
	10	1.27	0.0214	0.9703	0.9925	0.9973	0.9872
	12	1.15	0.0193	0.9732	0.9929	0.9973	0.9872
	14	1.24	0.0206	0.9720	0.9924	0.9975	0.9881
	16	1.10	0.0184	**0.9774**	0.9940	0.9975	0.9879
	18	1.12	0.0188	0.9755	0.9938	0.9975	0.9881
	20	**1.09**	**0.0183**	0.9760	0.9938	**0.9977**	**0.9888**
	22	1.16	0.0192	0.9752	0.9937	0.9974	0.9875
	24	1.13	0.0190	0.9763	**0.9941**	0.9970	0.9864
Sinusoidal pattern	4	1.55	0.0259	0.9592	0.9907	0.9960	0.9808
	6	1.34	0.0223	0.9678	0.9927	0.9964	0.9826
	8	1.39	0.0235	0.9662	0.9923	0.9966	0.9837
	10	1.31	0.0219	0.9684	0.9924	0.9970	0.9855
	12	1.29	0.0217	0.9688	0.9926	0.9974	0.9873
	14	1.15	0.0192	0.9759	0.9943	0.9971	0.9861
	16	1.17	0.0196	0.9723	0.9937	**0.9975**	**0.9880**
	18	1.17	0.0198	0.9736	0.9933	0.9973	0.9867
	20	**0.97**	**0.0163**	**0.9793**	**0.9951**	0.9970	0.9852
	22	1.18	0.0198	0.9762	0.9943	0.9973	0.9868
	24	1.19	0.0200	0.9789	0.9948	0.9974	0.9875

### 3.2 Validation Using Endoscope FPP System Data

**Fig. 7:**
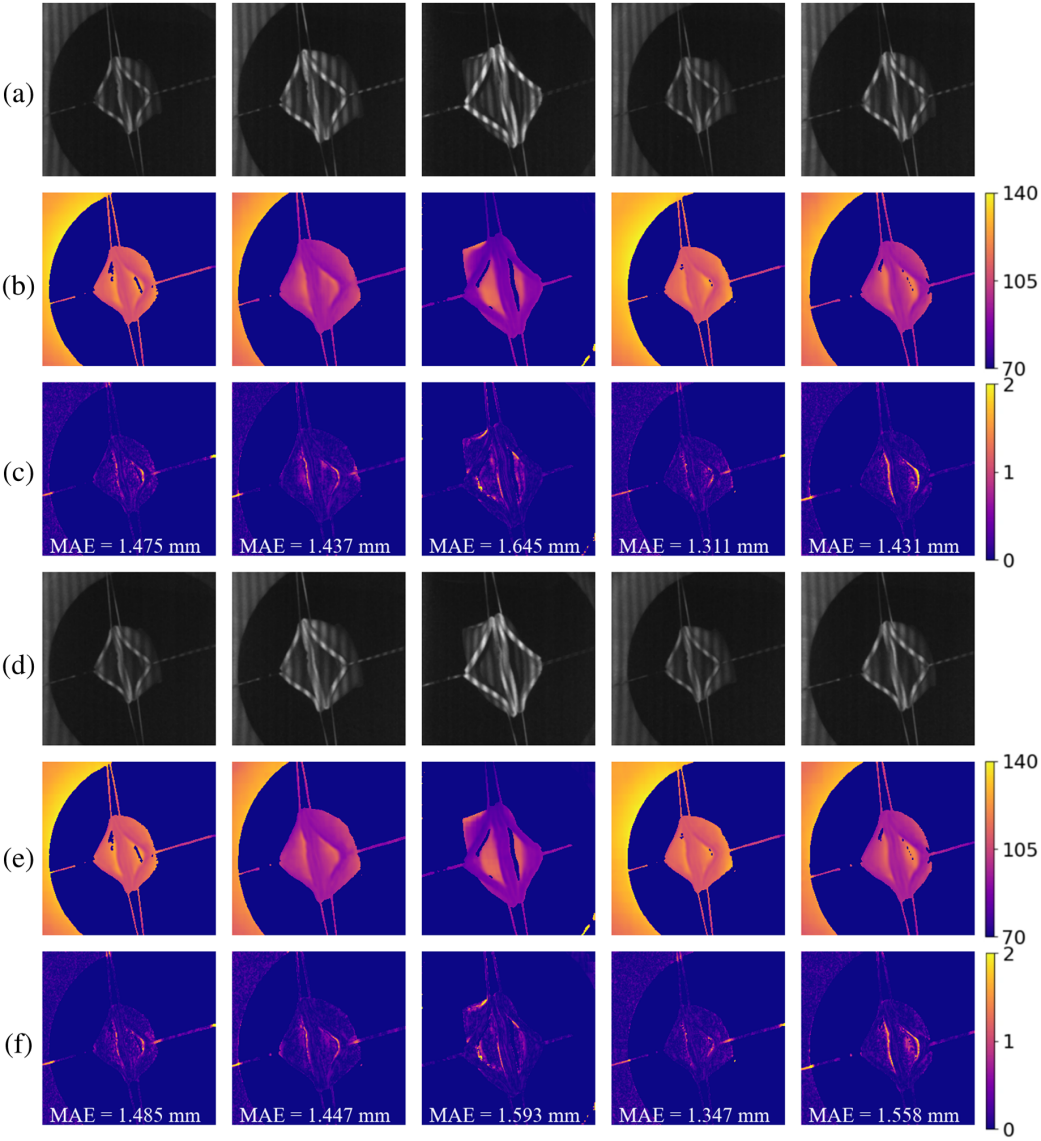
Depth reconstruction results and error maps across five example cases collected from the endoscope system. Rows (a) and (d) are original images with binary and sinusoidal patterns, respectively. Rows (b) and (e) are the corresponding depth reconstruction results. Rows (c) and (f) are error maps for comparison (depth unit: millimeter).

Based on the conclusions from the synthetic data, we conducted our endoscope FPP experiments using both sinusoidal and binary fringe patterns at 20 Hz. Figure 7 presents and compares the depth reconstruction results from both patterns across five example cases. Figure 7 rows (a) and (d) show the original images with binary and sinusoidal patterns, respectively. Rows (b) and (e) display the corresponding depth reconstruction results, whereas rows (c) and (f) present the error map and MAE for comparison.

We observed that the algorithm produces similar results for both types of fringe patterns. The ground truth, calculated using the conventional method, is highly sensitive to image intensity, leading to a noisy depth map. By contrast, the proposed method not only predicts the depth accurately but also generates a smoother image, demonstrating its robustness to low SNR images.

The detailed quantitative results are listed in Table 2. We observed that fringe images with both patterns produced similar results, with the binary pattern performing slightly better. To evaluate the performance differences between the two types of patterns, we conducted two-tailed paired t-tests on their respective metrics. All p values calculated were below 0.05, indicating that the depth reconstruction results of the binary pattern are statistically better. The images collected from the experimental setup often suffer from motion blur and low signal-to-noise ratio during real-time measurements. Consequently, the intensity of sinusoidal patterns is less accurate compared to synthetic data, resulting in higher depth prediction errors. By contrast, binary patterns are more effective in resolving phase ambiguities over larger depth ranges,^30^ leading to improved depth prediction accuracy.

**Table 2:** Quantitative evaluations on the experimental dataset with two patterns.

Pattern	Depth metrics				Segmentation metrics	
	MAE (mm) ↓	Abs Rel ↓	δ=1.1 ↑	$\delta =1.1^{2}$ ↑	DSC ↑	mIoU ↑
Binary pattern	2.28	0.0219	0.9726	0.9863	0.9944	0.9776
Sinusoidal pattern	2.38	0.0231	0.9716	0.9860	0.9950	0.9801

### 3.3 Algorithm Efficiency

The entire network has a parameter size of 175.42 MB, with DepthNet and MaskNet each using 87.71 MB due to their similar structures. The memory usage throughout the training process was 6.85 GB. To analyze GPU usage and explore the potential improvements in time consumption, we utilized PyTorch Profiler to obtain detailed execution and efficiency metrics. The results presented in Table 3, demonstrate that for a single frame depth prediction, the total processing time was 12.75 ms, where the GPU kernel, memory copy, CPU execution, and other operations accounted for 87.93%, 0.07%, 8.34%, and 3.66% of that time, respectively. Note that the acquisition time for each frame was 50 ms. The network depth prediction was faster than the image acquisition, and the system has the potential for acceleration through further optical optimization.

**Table 3:** Network execution time summary.

Category	Time duration (ms)	Percentage (%)
GPU Kernel	12.75	87.93
Memory copy	0.01	0.07
CPU execution	1.21	8.34
Other	0.53	3.66
Total	14.50	100

### 3.4 Ablation Study

**Fig. 8:**
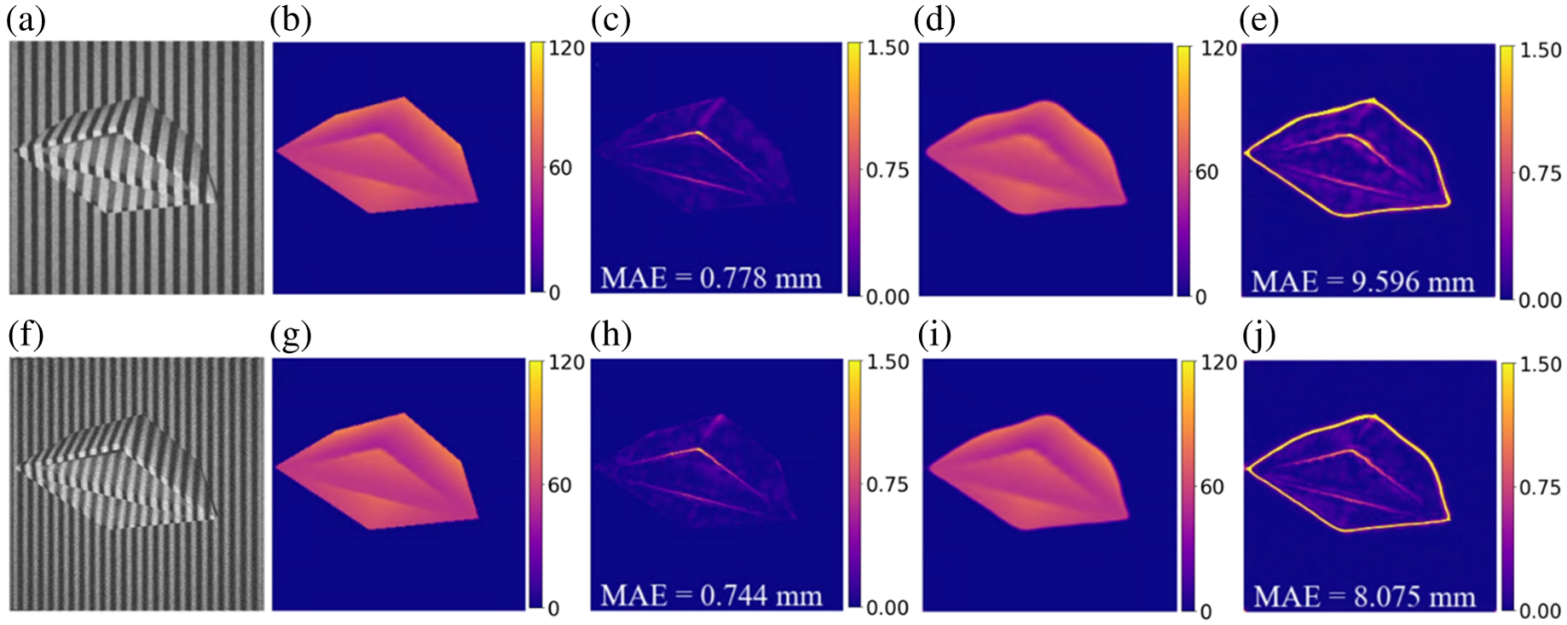
Depth reconstruction results and error map from (top) binary and (bottom) sinusoidal pattern image. Panels (a) and (f) are the original images with binary patterns and sinusoidal patterns, respectively. Panels (b) and (g) are the corresponding depth reconstruction results from the original network with MaskNet; (c) and (h) are the corresponding absolute error maps, respectively. Panels (d) and (i) are the corresponding depth reconstruction results from the network without MaskNet; (e) and (j) are the corresponding absolute error maps, respectively. Depth unit: millimeter.

To demonstrate the benefits of the two-path design of the network, we conducted an ablation study by removing the MaskNet. The synthesized dataset with 20 Hz sinusoidal and binary patterns was used for testing. The images were only fed into DepthNet for depth prediction training, with the same training strategies utilized as described in Sec. 2.7. The depth reconstruction results and corresponding error maps from network with and without MaskNet are shown in Fig. 8. Figures 8(a) and 8(f) show the original image with binary patterns and sinusoidal patterns, respectively. Figures 8(b) and 8(g) are the depth reconstruction results of the proposed method with MaskNet, whereas rows (d) and (i) are the depth reconstruction results without MaskNet, respectively. The corresponding error map and MAE of Figs. 8(b), 8(d), 8(g), and 8(i) are shown as Figs. 8(c), 8(e), 8(h), and 8(j). The results show that removing MaskNet leads to a noticeable decrease in depth-map prediction accuracy in both patterns. This is primarily because, without MaskNet, the network is required to also learn the depth prediction for background regions, especially those at the edge of the sample. Although the background in our dataset is relatively simple, it still introduces unnecessary complexity that can slow down the learning process and reduce overall accuracy. By explicitly segmenting the object of interest, MaskNet helps the network focus on relevant foreground features, thereby improving both training efficiency and prediction performance.

## 4 Discussion

In this paper, we successfully proposed and demonstrated an endoscopic single-shot fringe projection profilometry (FPP) system using a novel neural network designed to predict depth maps. The data acquisition system employed a dual-channel endoscope to project patterns and capture data through separate channels in one endoscope. The network was composed of two parts: the MaskNet to segment the sample and the DepthNet to generate the corresponding depth map. The network was trained and evaluated using both synthetic data and endoscopic FPP data. The system achieved real-time 20 FPS depth measurements within millimeter-level error using both binary and sinusoidal patterns.

Our novel approach is based on a dual-channel endoscope using a single-shot network algorithm. The conventional FPP system is constrained by its lengthy acquisition and processing times and requires a synchronization device between the projector and fringe patterns to align the camera exposure time with different fringe patterns. By contrast, the presented method eliminates the need for synchronization, reducing the system’s weight and complexity and facilitating easier integration with robotic systems.

The separate design of MaskNet and DepthNet was essential for the depth prediction task for several reasons. First, although the sample was easily distinguishable from the background in our experimental and synthetic data, real clinical scenarios have more complex backgrounds, where the texture and color may closely resemble the target tissue. An independent segmentation path is thus highly beneficial. The presented experiments primarily involved relatively simple tissue structures; the network’s ability to preserve the fine details of the tissue shows potential for generalizing to more complex surface topological structures. Second, using a single network would result in the error of training being dominated by background prediction, due to the large proportion of background pixels. Utilizing separate networks and loss functions helped our network focus on the depth prediction task for the sample as described in Sec. 3.4.

The conventional FPP based on a binary pattern mask with N stripes can encode the image up to $2^{N}$ combination of stripes in sequences.^17^ It is reliable to remove phase ambiguities, but less sensitive to surface characteristics than sinusoidal patterns due to the lack of a smooth phase gradient. Thus, the sinusoidal pattern is ideal for applications requiring fine surface detail. There is also a method that uses combined sinusoidal and binary patterns^31^ for accurate phase retrieval. In our proposed algorithm, the images of binary fringe patterns produced more accurate depth prediction. Because the pattern phase/intensity-depth mapping is highly sensitive to the image quality and motion blur, the binary fringe has the potential to be more robust for dynamic measurements of living tissue.

The proposed endoscope FPP optical setup can be considered to be an approximate coaxial structure. Its compact design minimizes optical aberrations, simplifies the mechanical layout, system calibration, and facilitates integration with robotic surgical systems as a 3D imaging system. However, one significant disadvantage of the system is that, due to space constraints, only a portion of the camera’s field of view is utilized, resulting in a lower numerical aperture and increased image noise. It may also decrease phase retrieval sensitivity to height variations compared to noncoaxial setups. This reduction in sensitivity can lead to slightly lower depth resolution. On the contrary, it improves the robustness of phase measurements against noise, due to the simplified mapping between image coordinates and surface points. In summary, our approximate coaxial system has a trade-off between sensitivity and robustness. Although it may slightly compromise depth sensitivity, it offers advantages in measurement stability and system alignment, particularly beneficial for real-time applications. Another key factor is that the ground truth is generated using the conventional FPP algorithm, which relies on the image quality from the camera. The non-uniform lighting could distort the fringe patterns and reduce the phase estimation accuracy and limit the network’s prediction capabilities. Several optimizations can be made to further improve the optical setup. The DMD chip in the projector adopts a diamond layout and exhibits diagonal flipping behavior, which can introduce spatial non-uniformity and aliasing artifacts. These effects often lead to fringe distortion and phase retrieval errors. To address these issues, we implemented the following strategies. First, we applied low-pass filtering and bicubic downsampling to the input image to suppress aliasing from the diamond grid while preserving the necessary fringe frequencies. Second, our single-shot network employed binary patterns, which inherently avoid phase noise induced by mirror flipping. This is because the DMD mirrors operate in fully ON/OFF states, eliminating partial-angle settling artifacts and ensuring both intensity uniformity and temporal consistency, which are critical for accurate phase and height retrieval. Moreover, by leveraging the single-shot network, in the future, we can replace the projector with a simple LED light source and a fixed binary mask to generate the required fringe patterns. This substitution will eliminate the projector’s influence entirely, further improving system stability and performance. In addition, the decreased size of the illumination section could save space for the camera and provide for better image quality, also allowing for more adaptive optical configuration.

In addition, the accuracy discrepancy between the synthetic and experimental data stems from the differing methods of ground truth generation. Using a more accurate approach, such as a computed tomography^32^ or optical coherence tomography^33^ system, could significantly improve the results. Due to the limitation of projector intensity, the acquisition time for each frame was 50 ms (20 FPS), which is a longer exposure time than what is achievable otherwise. The algorithm achieved a processing time of 12.75 ms (≈78 FPS) for each frame. A higher frame rate is feasible with improvements in the light source and optical design.

The current experiments focused on intestinal tissue imaging due to its relevance in task-specific, constrained surgical environments where rapid and compact 3D sensing is essential.^4^ In clinical scenarios, more complex tissue structures and various forms of tissue deformation may pose challenges to both depth prediction and tissue segmentation. To enhance clinical applicability, future studies will focus on extending the system to a broader range of anatomical structures, such as layered tissues.^34^

## 5 Conclusion

In summary, we described an endoscopic single-shot FPP system to achieve real-time depth map generation using a deep learning network. The network was tested on both synthetic and our experimental phantom sample data to assess model generalization. The system was evaluated using both the binary and sinusoidal fringe patterns. Both patterns achieved an absolute depth measurement error less than ∼2  mm (absolute relative error 2%∼3%) at an acquisition and processing speed of 20 frames per second, thus establishing the feasibility of a low-cost single-shot system based on a binary pattern.

## Data Availability

Available upon reasonable request to the corresponding author.
